# Restrictions on the Practice of Physicians

**Published:** 1811-04

**Authors:** 


### To the Editors of the Medical and Physical Journal.

GENTLEMEN,

DURING the present arduous contest for the vacancy in St. Luke's hospital, occasioned by the resignation of Dr. Simmons, I have heard much of the necessity that a physician who undertakes the important charge of the patients in that hospital, should be practically conversant with the treatment of insanity in particular. I admit that in some respects this would be an advantage, but I cannot regard it as an essential one. I ask, is there any one regularly educated physician, accustomed to see and to treat diseases in general, who, if suddenly called for the first time to attend an insane patient, would be so ignorant of the nature of mental derangement, as to be at a loss how to act? I confess that were such a practitioner pointed out to me, I should pronounce him unfit to pursue a profession which demands a knowledge of every variety of disease. The popular cry is in favour of practitioners devoting their attention to one disease in particular, and so well has this been seconded by interested individuals, that the physician is now excluded from treating many complaints that formerly were in his immediate province. Thus he has lost all influence over complaints of the eye and the ear; he is not suffered to treat any of the various forms which syphilis assumes; scrofula is no longer under his control; it is now the fashion to consign the diseases of women and children to the diplomatized accoucheur, and whilst the physician very properly refrains from interfering with the chirurgical department, he hears of *chaste* surgeons treating continued fever, pulmonary consumption, and, in short, any other general disorder to which the frame is incident; and their *prescriptions* too ratified with their signatures. Without wishing to excite insidious jealousy between the two branches of the profession; I would call the attention of the readers of your Journal to this rapidly growing *stricture* of the physician's practice in London. Besides the undue and unprofessional encroachment of the surgeon on the function of the physician, he is elbowed out of practice by the apothecary, who in turn is shuffled off by the druggist. Gentlemen, that acute observer, Dr. Beddoes, remarked, that the time was approaching when there would be fewer physicians, meaning that the public would become too enlightened to require so much of their aid; I think that the time has arrived when, for their own interests, there ought to be fewer. So far from this diminution being effected by the superior wisdom of the public, it would seem to be accomplished by its folly; a more expensive, and at the same time a more inferior and dangerous species of medical assistance being substituted for that afforded by the regular physician, who is now in very many instances the last person, except the undertaker, thought of to be introduced into the sick man's chamber. At what period in the annals of medicine has quackery been more triumphant? when have the chariots of illiterate pretenders to the art more abounded, or when have there been a greater number of deserving physicians unemployed? Is this then a time to encourage the belief that a regular physician is not to be entrusted with the treatment of insane persons, as well as with that of any other patient affected with general disease? Unfortunately the malady is usually so little under the influence of medicine, that the keepers have much more to do with the patient than the physician has; and I am at a loss to comprehend how evacuants will operate more effectually when directed by a *mad* doctor, than when prescribed by an ordinary physician; nay, I will venture to assert, that in many instances there is danger of establishing a disease from the dread inspired by a *mad* doctor, which would not have occurred had a common practitioner been consulted. By avoiding restraint and using soothing means, we sometimes avert the threatening malady.

"The

"——— the shrewd doctor, in the spleen-struck mind
When pregnant horror sits, and broods o'er wind,
Discarding drugs, and striving how to please,
Lures on insensibly, by slow degrees,
The patient to those manly sports, which bind
The slacken'd sinews, and relieve the mind:
The patient feels a change as wrought by stealth,
And wonders on demand to find it health."

In the hopes that some more able correspondent will pursue this interesting subject,

I am, Gentlemen,

Your's, &c.

CENSOR.

*London, March* 4, 1811.

